# Emigration as a social and economic determinant of health in low-income urban Zimbabwe

**DOI:** 10.1186/s41043-022-00330-w

**Published:** 2022-11-05

**Authors:** Tafadzwa C. Chigariro, Marvellous M. Mhloyi

**Affiliations:** Centre for Population Studies, University of Zimbabwe, Harare, Zimbabwe

**Keywords:** Emigration, Remittance, Health, Social Determinants of Health, Nutrition

## Abstract

**Background:**

The negative consequences of medical professionals’ emigration on the health systems of nations are well documented in the literature. However, there is a dearth of evidence on the impact of emigration in general, on sending households’ welfare, health in particular. This study compared socio-economic characteristics, including health, of emigrants’ households with those of non-emigrants’ households in an urban setting in Harare, Zimbabwe.

**Methods:**

A cross-sectional survey and focus group discussions were used to collect quantitative and qualitative data, respectively. Concurrent and retrospective data were collected using an interviewer-administered questionnaire. The target population were households, both emigrants' households and non-emigrants' households, and the interviewees were de facto heads of the respective households.

**Results:**

A sample of 279 households was determined; however, 268 heads of households, a response rate of 96%, were achieved. The majority of the respondents were females (52%). Emigrants’ households were more likely to access private compared to government health care facilities, than non-emigrants’ households [*P* = 0.001]. Emigrants' households were also more likely to report higher incomes than non-emigrants’ households [*P* < 0.05] and were having more meals per day and better access to education. Emigrants' households were also more likely to report positive lifestyles than non-emigrants’ households. Only 13.8% of emigrants' households reported a negative shift in lifestyle, compared to 25.2% non-emigrants' households.

**Conclusions:**

Emigration was found to have a positive relationship with health seeking, income, education, and number of meals a household had. It is clear from the findings that emigration during the hard economic times in Zimbabwe is beneficial; it cushions households from the ravages of poverty. Yet emigration robs the nation of its professional able-bodied people. It is, therefore, recommended that the government optimises the reported positive effects, whilst expeditiously working on improving the economy with the view of reversing the observed migration streams.

## Introduction

Migration has become a global phenomenon, touching nearly all corners of the world [1]. Yet, the effects of migration are increasingly becoming multifaceted, both at places of origin and at destinations [2]. Migration itself is a product of factors that help keep a migrant in their home area (push factors) and those that drive a migrant from the home area (pull factors) [3, 4]. Push factors include non-availability of economic and social opportunities, poverty, insecurity, overpopulation, poor living conditions, desertification, famines/droughts, fear of persecution, poor health care, loss of wealth, and natural disasters [5]. Pull factors include better job prospects, better living conditions, political freedom, better education facilities and assisted welfare systems, better commuting networks and communication facilities, better health care systems, better security and equality before the justice system [5].

The impact of emigration (out-migration) appears to be framed by two extremes. In some sending areas, migration has set in motion a development force, as remittances facilitate various kinds of investment. This includes direct and indirect investment in health and health care activities, including better access to essential treatment. In some cases, emigration has, however, drained local economies and societies of their human and financial capital [6]. Yet, studies on the relationship between emigration and health at the place of origin have been limited to the attrition of skilled health professionals through migration [7, 8].

Studies elsewhere, have noted migration, remittances in particular, to have favourable impacts on health outcomes [9–12]. This is because the additional income coming from remittances increases the ability to access health services, buy expensive medicine, and eat better-quality food [9, 10]. Adhikari et al. observed that elderly persons with migrant children were more likely than those whose children had not migrated to seek treatment, after controlling for socio-demographic and economic variables [10]. Remittances have also been associated with improved housing and water and refrigeration of food [13], among other determinants of health. For instance, adults in emigrants’ households were noted to be significantly less susceptible to being underweight than those in non-migrants’ households, yet they did not have an increased risk of being overweight [11]. However, migration was associated with increased chances of having symptoms of poor mental health among elderly persons residing in the emigrants’ households [10]. Given that remittances increased from US$ 36.9 billion in 2014 to US$ 39.8 billion in 2015, representing 2.16% and 2.59% of GDP for the respective years [9, 14], their relationship with health and health outcomes requires more attention.

Zimbabwe endures a very high migrant stock, with UNDP estimating 3.5 million of the population to be living in the diaspora [15]. Ratha et al. place the net migration for Zimbabwe at 11.1 migrants per 1000 population, translating to a migrant stock of over 4 million [16]. This means about a quarter of the Zimbabwean population is in the diaspora. The high level of emigration is associated with crippling, sometimes permanent, skills losses in the health and other sectors. This notion, however, overlooks the role of remittances as a determinant of health at the place of origin. Skeldon contends that remittances have a positive impact on the place of origin [17]. Recruitment and Returns are other key dimensions of migration, with an impact on health, that is worth considering. Recruitment deals with the employment status (employed, unemployed or underemployed) of migrants on departure and at destination. Returns refers to migrants who come back to their countries of origin. The Government of Zimbabwe thus recognises the importance of remittances as an economic driver. However, whilst diaspora remittances to the country have increased from 294 million (US$) in 2009 to 935 million in 2015, they have since been on the decline, reaching 635.43 million in 2019 [18].

The current economic situation in Zimbabwe is forcing people to emigrate in the hope of securing employment in countries with better economies such as South Africa, Botswana, UK and Australia [19]. The economic challenges are characterised by high unemployment rates, inflation and low productivity [20]. Those who migrate, however, may not get a job in their destination countries as soon as they would have anticipated. They may also find it difficult to have their qualifications recognised in the countries of destination. As a result, they end up in a worse economic situation than they were before they migrated. Considering that those with a high tendency to migrate tend to be breadwinners in their families, the situation may be worse for those they leave behind. When this happens, the health and general socio-economic situation of the families left behind is also affected.

During the 1980s and most of the 1990s, the government of Zimbabwe invested in primary and preventive health care and rolled out primary health care services to within 10 kms of at least 80% of the population [21]. However, the health delivery system has deteriorated with shortages of skilled professionals and health care staff, a lack of functional equipment, and a lack of essential medicines and commodities becoming persistent [22]. Disruptions and strikes by health care professionals over wages and working conditions and rampant corruption have also become a major problem [23]. Zeng et al. further notes that this deterioration coincided with a fall in demand for services, following the introduction of user fees in public health facilities, which are often applied in an ad hoc way and have been noted to drive households into poverty [21]. Consequently, demand for private health care in Zimbabwe has surged [24], despite the costs involved. This is within a context where universal health insurance is non-existent and only 10% of the population has medical aid cover [24].

It is also important to note that Zimbabwean suburbs (urban districts) are highly structured and homogenous [25]. This is a legacy of the colonial history of the nation, which saw neighbourhoods being organised around race, colour, social standing and employment/economic status [25]. Hatcliffe District—a low socio-economic status community—is therefore expected to host households with similar social status and economic standing, with minimum deviations.

This study explores the economic impact of emigration on health by comparing access to health and health care among emigrants' households and non-emigrants' households in urban Zimbabwe. It also explores the contribution of emigration in shaping other social determinants of health such as education and household nutrition security.

## Methods

This study extensively drew from Frankenberger’s ‘Household Livelihood Security Framework [26]. The ‘Migration and Household Livelihood Security Framework’, as adapted from Frankenberger and McCaston, defines household livelihood security as adequate and sustainable access to income and resources to meet basic needs such as adequate access to health facilities, food, potable water, educational opportunities, housing and social integration [26]. In the absence of theoretical frameworks on migration and the determinants of health, the study charted the pathways in which (a) migration and remittances directly influence the (b) socio-economic determinants of health identified by Frankenberger (economic security, adequate access to food, potable water, health facilities, educational opportunities, housing and social integration) and how it indirectly affects (c) health outcomes at the household level. Study questions were then framed around the three levels (a–c) of the adapted framework described above. The broad questions included an evaluation of the economic impact of emigration on health and how emigration shape other social determinants of health such as education and household nutrition security among emigrants' households and non-emigrants' households, respectively. Access to health and basic education as well as shifts in healthy lifestyles were evaluated by asking interviewees to rate their households’ situation on a Likert scale (poor–very good).

The study setting is Hatcliffe District, a low socio-economic status and expanding high-density suburb about 25 km out of Harare North District, in Harare, Zimbabwe. The study design was a mix of quantitative and qualitative methods to allow for a complete analysis of both numerical and contextual data. A cross-sectional survey was employed to collect concurrent and retrospective data.

Consistent with similar studies, the sampling unit was a household [27, 28]. The studies emphasise that the ‘household’ is the socio-economic unit, whilst the ‘family’ primarily is by reference to relationships which pertain to or arise from reproductive processes and which are regulated by law or by custom. This definition is more relevant in African contexts (Zimbabwe in particular), where many households include *de jure* members with a different relationship to the head of the household. Both emigrants' households and non-emigrants' households were included to allow for comparison between the households. Emigrants' households were defined as those from which one or more permanent member(s) had relocated to another country permanently for a period over six months preceding the study [29].

Multistage random sampling was employed in the study. Hatcliffe District was randomly selected from a list of all high-density suburbs in Harare. Nine enumeration area (EA) maps showing the location of households and major population points in Hatcliffe were then obtained from ZIMSTAT. The EAs were selected using probability proportionate to size (PPS) sampling (using a computer-based system). This approach ensured that households in larger EAs had the same probability of getting into the sample as those in smaller EAs, and vice versa. A total of 31 households were selected from each EA. A sampling frame was then obtained from the selected EA maps and simple random sampling was employed to select the study units within the selected EAs. The interviewees were the de facto heads of all the selected households in Hatcliffe District on the date of the interview.

An Interviewer Administered Questionnaire developed based on standard questionnaires used in previous studies by IOM was used for data collection. The questionnaire included pre-coded and open-ended questions on the impact of emigration on household livelihood. Statistical Package for Social Sciences (SPSS 17) was used to analyse the data. Data analysis involved running of summary measures or descriptive statistics as well as inferential statistics. Multinomial logistic regression models built using the NOMREG function in SPSS were also used to analyse the associations between the variable, ‘where interviewed households last accessed health care’ when their member fell ill and the predictor variables, ‘household emigration status’ and ‘average household monthly remittances’. Results with a *P* value of =  < 0.05 were considered statistically significant. The outputs were presented in graphs, charts and cross-tabulations.

Focus group discussions were conducted to triangulate the quantitative findings. Three focus group discussions were conducted at the major shopping centre and at two local churches in Hatcliffe, respectively. These were deemed to be the central sites of the community. This qualitative method was used to provide a narrative description of the data obtained from the quantitative methods. It answered the questions: why? how? and in what way? to yield opinions, experiences and feelings of individuals. A thematic approach was taken in analysing the information gathered through focus group discussions and the opinions expressed were presented to support quantitative findings.

## Results

A target of 279 respondents, de facto household heads, were selected out of which 268 were successfully interviewed (see Table 1). This yielded a response rate of 96%. The majority of the respondents were female (52%). Reported emigration levels were quite high, with 110 (41%) out of the 268 interviewed households reporting emigration (see Table 2). Less than 60% of the households did not report emigration. Overall, 151 persons had emigrated from the surveyed households. This yields an average of 108 emigrants per 1000 population. Those who emigrated were more likely to be heads of households, that is fathers (46%) and mothers (44%).

**Table 1:** Percentage distribution of demographic characteristics of respondents (*n* = 268)

	Percent (%)
Age group of respondent	
16–19	7.8
20–29	32.8
30–39	25.7
40–49	14.6
50–59	5.6
60 +	13.4
Total	100
Sex of respondent	
Male	47.8
Female	52.2
Total	100
Position in household	
Father	45.9
Mother	44.0
Child	4.5
Grandmother/other relative	5.6
Total	100

**Table 2:** Percentage distribution of emigration for interviewed households (*n* = 268)

	Percent (%)
Household emigration status	
Emigrants' households	40.7
Non-emigrants' households	59.3
Total	100

South Africa, UK, Botswana and Malawi were the most cited destination countries for emigrants from Hatcliffe. The largest proportion of emigrants, 51%, was in South Africa. Another 11% of emigrants were in the UK, while Botswana and Malawi had each 8% of the emigrants. Only 3% of the emigrants were in Namibia, whilst other European countries and Asia were home to 5% of the emigrants each.

**Fig. 1:**
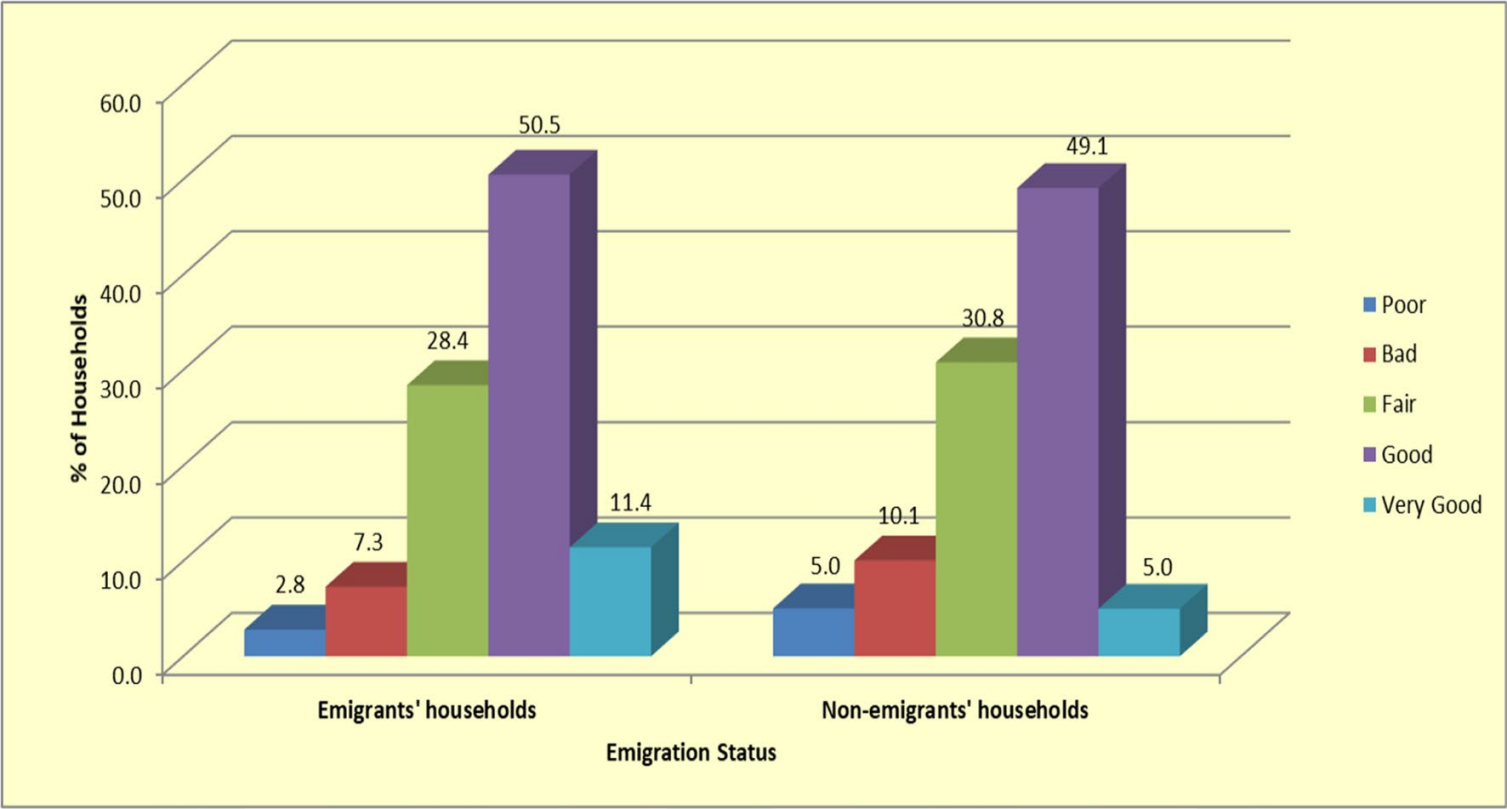
Percentage distribution of ratings of household access to health facilities for emigrants' and non-emigrants' households (*n* = 268)

Emigration was associated with better access to health and health care facilities. For instance, emigrants' households were more likely to report better access to health services when compared to non-emigrants' households. At least 61% of the emigrants' households rated their access to health facilities to be at least good, compared to 54% of non-emigrants' households (See Fig. 1). In addition, emigrants' households were more likely, to report that they had sought treatment the last time a member of the household fell ill compared to non-emigrants' households (Pearson chi-square test of association [*χ*^2^(2) = 1.751 (*P* < 0.05)]) (see Table 3).

**Table 3:** Cross-tabulation of household emigration status and treatment seeking (*n* = 268)

	Was treatment sought			Total
	Yes	No	Not applicable	
HH migration status				
Emigrants' households				
Count	82_a_	19_a_	8_a_	109
% within emigrants' HH	75.2%	17.4%	7.3%	100.0%
Non-emigrants' households				
Count	117_a_	23_a_	19_a_	159
% within non-emigrants' HH	73.6%	14.5%	11.9%	100.0%
Total				
Count	199	42	27	268
% within HH	74.3%	15.7%	10.1%	100.0%

Each subscript letter denotes a subset of ‘Was treatment sought’ categories whose column proportions do not differ significantly from each other at the .05 level

Results of nominal regression models assessing cross-sectional associations between the dependent variable ‘where interviewed households last accessed health care’ (i.e. private health facilities, council/government facilities or other facilities) and the predictor variables, ‘household emigration status’ and ‘average household monthly remittances’, indicated the associations to be significant [*P* = 0.000]. This proves that these predictors contribute significantly to the final model (See Table 4). In addition, the adjusted ORs show that emigrants’ households were 12.314 times more likely to access a private health care facility compared to a local government (i.e. council)/government health care facility than non-emigrants’ households [*P* = 0.001]. Adjusted ORs also show that emigrants’ households are 5.074 times more likely to access a mission hospital or a private pharmacy (other facilities) than a government facility [*P* = 0.05]. While remittances were a significant predictor variable for the overall model [*P* = 0.000], the actual values of average monthly remittances received by emigrants’ households did not seem to significantly influence households’ ability to access the different types of health care facilities assessed. The duration of stay abroad of respective family members was also not a significant predictor variable.

**Table 4:** A multivariable logistic regression on predictors of access to private, government and other health care facilities in low-income urban Zimbabwe (*n* = 200**)

Characteristics	Private health facilities			Other facilities			Council/government facilities		
Emigration status	Count	Adjusted OR	95% CI	Count	Adjusted OR	95% CI	Count	Adjusted OR	95% CI
Emigrants' households	25	12.314*	2.82, 53.775	14	5.074*	0.983, 26.199	25	1	1
Non-emigrants' households	21	1	1	27	1	1	88	1	1
Average monthly remittances (US$)									
0	23	7128E7	.000, .c	26	4991E7	.000, .c	80	1	1
50	3	1.505E16	.000, .c	0	3113E7	3113E7, 3113E7	0	1	1
60	0	1	.000, .c	0	1	.000, .c	4	1	1
90	0	1	.000, .c	0	1	.000, .c	4	1	1
100	0	2386E7	2386E7, 2386E7	4	1.848E16	.000, .c	0	1	1
120	4	6.693E14	.000, .c	3	5.741E14	.000, .c	0	1	1
130	0	1.153	.000, .c	0	1.102	.000, .c	3	1	1
200	4	3863E7	.000, .c	0	4.658	.000, .c	4	1	1
220	4	1937E7	.000, .c	0	2.067	.000, .c	7	1	1
230	0	1.153	.000, .c	0	1.102	000, .c	3	1	1
250	0	2386E7	2386E7, 2386E7	4	1.848E16	000, .c	0	1	1
300	0	3.246	.000, .c	0	3.232	000, .c	4	1	1
400	1	2.911E14	.000, .c	0	2239E7	000, .c	0	1	1
500	3	2.175E14	.000, .c	4	3.092E14	000, .c	0	1	1
960	4	1.222E16	.000, .c	0	2720E7	2720E7, 2720E7	0	1	1
1000	0	2278E7	.000, .c	0	2147E7	000, .c	0	1	1
1393+	0	.1	.1	0	.1	.1	4	1	1

*Statistically significant (*P* value =  < 0.05); 1: Reference category; **Response category Not applicable is omitted

**Fig. 2:**
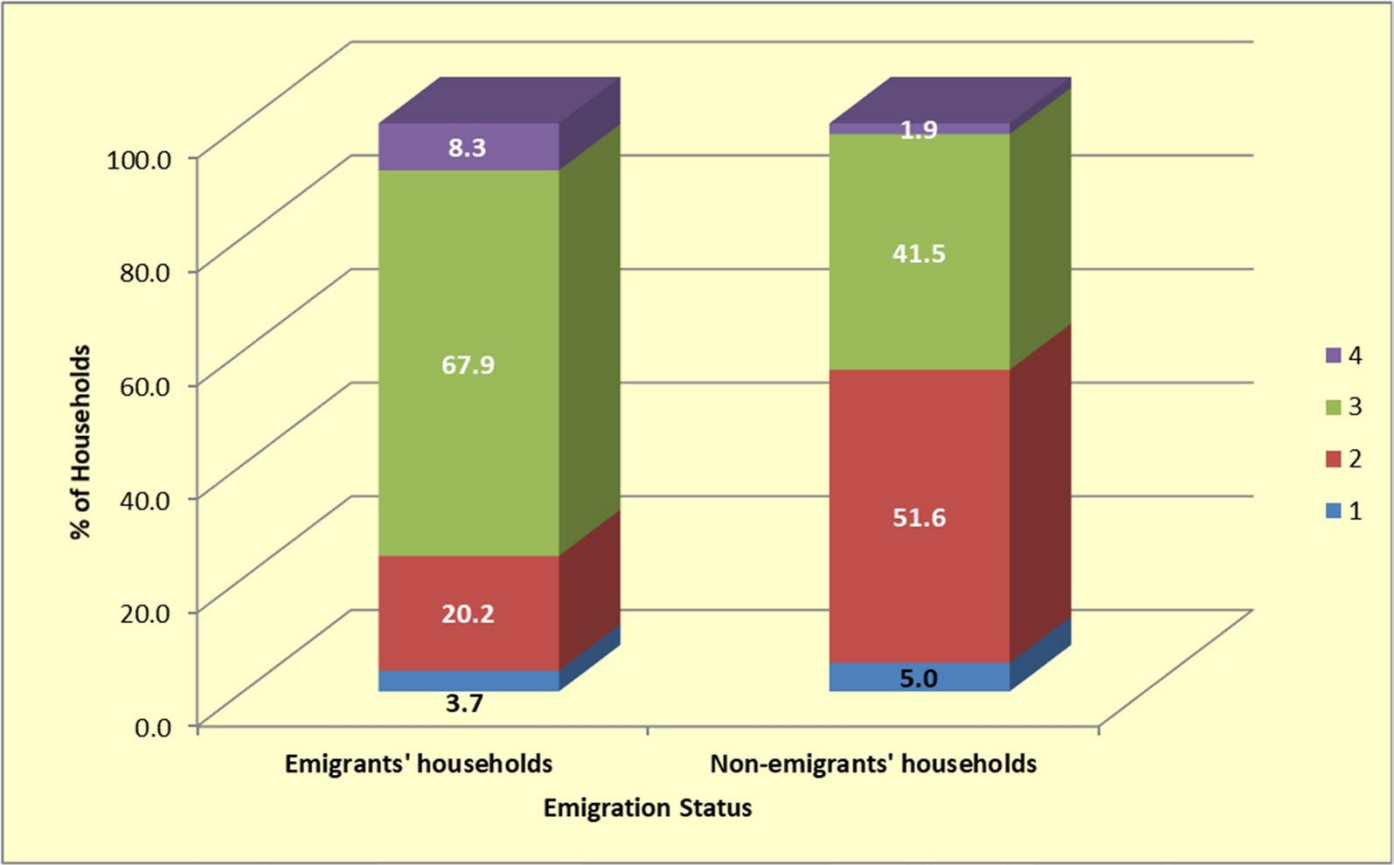
Percentage distribution of number of meals eaten daily for emigrants' and non-emigrants' households (*n* = 268)

Follow-up questions revealed that emigrants' households maintained healthier diets compared to non-emigrants' households. About 68% of emigrants' households reported having at least three (3) meals per day compared to only 42% of the non-emigrants' households (See Fig. 2). Emigrants' households were also more likely to maintain positive lifestyles. Only 13.8% of emigrants' households reported a negative shift in lifestyle, compared to 25.2% of the non-emigrants' households group (See Table 5).

**Table 5:** Percentage distribution of reported change in household lifestyle for emigrants' and non-emigrants' households (*n* = 264)

	Positive	Negative	No change	Total
HH migration status				
Emigrants' households	62.4	13.8	23.9	100.0
Non-emigrants' households	53.5	25.2	21.3	100.0

**Fig. 3:**
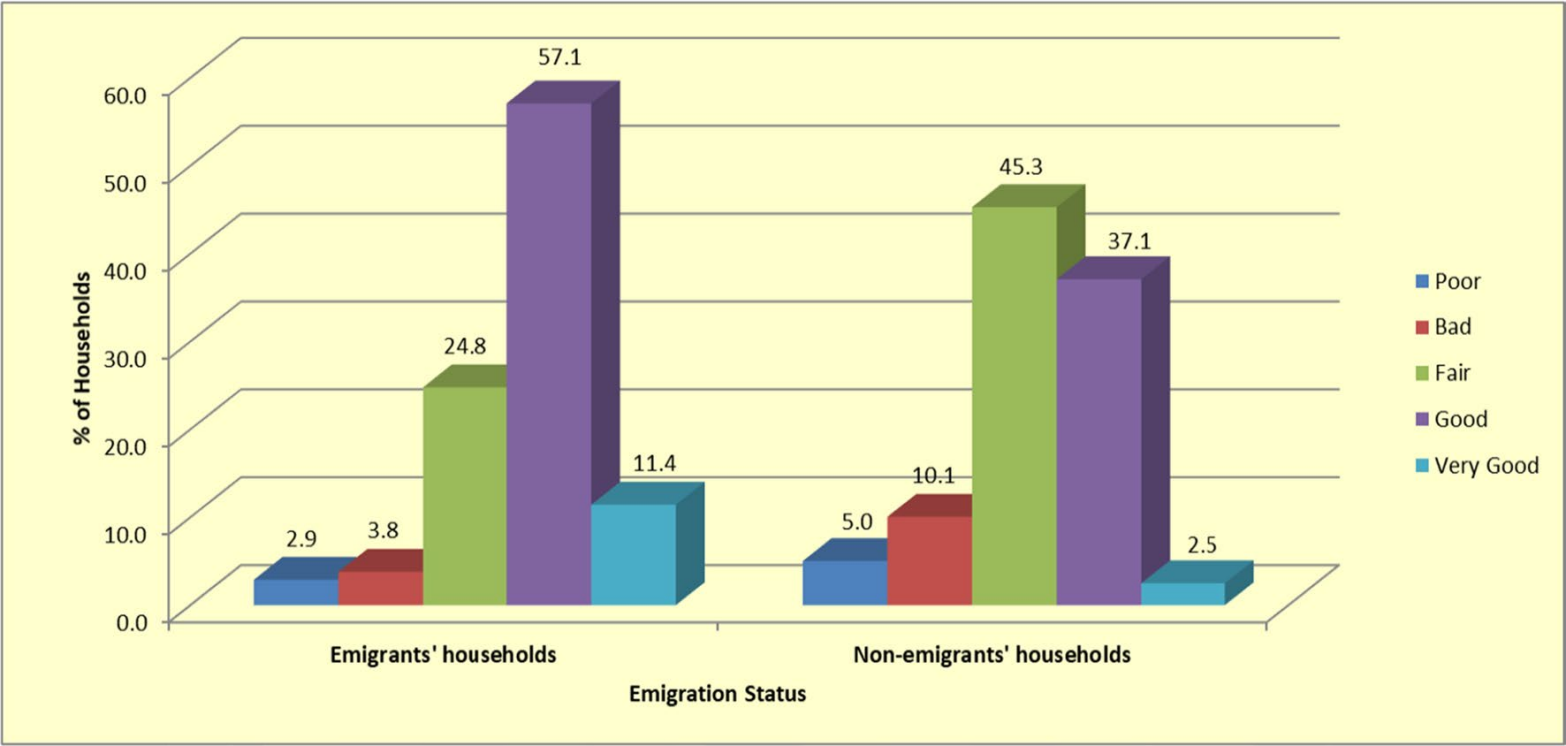
Percentage distribution of ratings of household access to education for emigrants' and non-emigrants' households (*n* = 268)

Emigration was also associated with better access to education opportunities by household members at the place of origin. Approximately 69% of the emigrants' households rated their access to education opportunities to be at least good, compared to only 40% of the households that had no emigrants (see Fig. 3).

Study participants who participated in FGDs concurred that remittances were a key and reliable source of income for financing the education of household members. They noted that emigrants viewed financing the education of members at places of origin as a way of creating lasting solutions to the burden of perpetually sending money home, as educated member would later either secure jobs locally or also migrate resulting in a shared burden of taking care of the household members at the places of origin. Participants further acknowledged the opportunities education brings to the household through improved health literacy and better prospects to earn income, which could also be used to finance health care and, health and its determinants.

Data from the study also tend to suggest that emigration has a positive impact on household income (Pearson chi-square test of association [*χ*^2^(4) = 12.3 (*P* < 0.05)]). Emigrants' households had a mean monthly income of the USA $530.56 compared to $477.31 in non-emigrants' households (See Table 6). This is against an overall mean income for the study population of $523.95 and an overall income range of between $20 and $2,500. Further, 30% and 38% of the emigrants' households had incomes in the ranges of $150–$300 and $301–$600, respectively, compared to 21% and 33% of the non-emigrants' households, respectively. While 21% of non-emigrants' households had incomes less than $150 in the 6 months preceding the study, this compares to only 6% of the emigrants' households. The income gap, however, seems to narrow down as household incomes approach $1,200. About 19% of the non-emigrants' households had incomes in the range of $601–$1200, compared to 18% of emigrants' households who had incomes in the same range.

**Table 6:** Distribution of household monthly incomes for emigrants' and non-emigrants' households (*n* = 268)

	Average HH monthly income category					Total
	< 150	150–300	301–600	601–1200	1200+	
HH migration status						
Emigrants' households	7	33	42	20	7	109
Non-migrants' households	34	34	53	30	8	159
Total	41	67	95	50	15	268

It was clear from the FGDs that the community perceive emigration as the only viable option and answer to the economic and health-related challenges that most people in the area suffered. Participants remarked that in their community, households with emigrants are better off, with the construction of most of the new buildings in the local being financed by remittances; households with emigrants have better and adequate food, better access to medical facilities and better clothing. They maintained that emigration is just the answer to the social and economic problems in Zimbabwe these days.

The relative importance of remittances as a source of household income had also not changed over the years. For instance, in both 2010 and 2011 remittances were the third most significant source of household income, contributing 12% and 16%, respectively (See Table 7). Labour and self-employment were the first and second sources of income in 2010 and 2011, contributing 34% and 31%; and 22% and 23%, respectively. Formal employment levels were, however, low, with only 33% of the population being employed. Self-employment was another major source of household income, contributing 22% in 2010 and 21% in 2011, respectively.

**Table 7:** Percentage distribution of sources of household incomes over years (*n* = 268)

	Reference period		
	2010 (%)	2011 (%)	Change (%)
Gifts	6.7	6.7	0
Cash transfers	0.9	0.9	0
Remittances	12.4	16.5	4.1
Petty trade	11.9	11.8	− 0.1
Self-employment	21.9	21.7	− 0.2
Livestock sales	0.7	1.5	0.8
Crop sales	6.7	4.1	− 2.6
Labour	34.3	31.3	− 3
Other	4.6	5.6	1.0
Total	100	100	3.6

## Discussion

The study confirmed the generally large migrant stock for Zimbabwe [30], but maintained a median locus. The estimated migrant stock of 108 emigrants per 1000 population (11%) is comfortably between internationally and nationally reported estimates, both of which are potentially motivated by economic ambitions. It is lower than the reported international average of 27% or 270 emigrants per 1000 population, but higher than the conservative average national stock reported by the Government of Zimbabwe of (361,743 emigrants) 2.9% or 29 emigrants per 1000 population [15, 19, 31]. However, it is agreeable that the migrant stock for the country is high, making this a good setting for studies to assess the impact of emigration on health at the place of origin.

It is arguable that emigration had a positive socio-economic—including health—impact on households in a low-income urban area in Harare, Zimbabwe. This follows the reported impact of emigration on the study households’ income (Pearson chi-square test of association [*χ*^2^(4) = 12.3 (*P* < 0.05)]). Studies elsewhere have observed that income is associated with better health outcomes, as households and individuals are better able to purchase health care and access other determinants of health such as good nutrition and better living conditions [32, 33].To further confirm this notion, the survey findings showed that emigrants' households were more likely to seek treatment the last time a member of the household fell ill (Pearson chi-square test of association [*χ*^2^(2) = 1.751 (*P* < 0.05)]). Furthermore, when given a choice between private, other (i.e. mission hospitals and private pharmacies) and government health care facilities, emigrants’ households reported better chances of accessing private health care facilities [Adjusted OR 12.31 (*P* = 0.001)] and other facilities [Adjusted OR 5.074 (*P* = 0.05)], respectively, compared to non-emigrants’ households. This difference in the ability to access better-quality health care services observed between emigrants’ households and non-emigrants’ households can be attributed to the role of remittances [*P* = 0.000], however, notwithstanding the actual value of remittances received by emigrants’ households. These findings are consistent with conclusions from studies elsewhere that remittances have a significant and positive effect on health expenditure, increase health expenditure across quintiles and the effect is substantially larger than the effect of income [12]. The fact that 61% of emigrants' households rated their access to health facilities to be at least good, compared to only 54% of non-emigrants' households further validates the notion that emigration is associated with better health. Chakraborty (2003) reported similar findings in the Journal for Health Promotion International [34].

The study findings also consistently show that emigrants' households were generally more likely to report positively on social and economic determinants of health status, compared to non-emigrants' households. For instance, emigrants' households enjoyed better nutrition and were more likely to engage in positive lifestyles. About 68% of emigrants' households, for instance, reported having at least three (3) meals per day compared to only 42% of the non-emigrants' households. This finding is also consistent with studies conducted elsewhere [35, 36]. Education is another key determinant of health, with past studies associating higher educational status with better health outcomes [37]. Given that nearly 69% of the emigrants' households group rated their access to education opportunities to be at least good compared to only 40% in the non-emigrants' group, it can be argued that emigration is a key determinant of access to education, and in turn has a positive effect on health in low-income urban areas.

Furthermore, the study shows remittances to be a resilient contributor to household income. The study findings indicate that the relative importance of remittances as a source of household income had not changed over the years, with remittances remaining the third significant source of household income in 2010 (12%) and 2011 (16%), respectively. Pant and Solimano have in separate studies, similarly reported remittances to be a stable source of income for households [38, 39]. We therefore infer that the impact of emigration and remittances on health in similar settings is long term.

The evidence suggests that emigrants’ households receiving remittances are protected against health shocks whilst on the contrary, households not receiving remittances or social assistance, find themselves vulnerable when their member fall sick [40]. Governments in developing countries should therefore investigate and account for the role of remittances when developing universal health care coverage policies, to ensure that both the poor and rich are protected against health shocks and to reduce health inequality.

In the absence of universal health insurance schemes in most developing countries [40], governments should partner with the private sector in harnessing remittances as a way of financing health insurance schemes through availing lucrative packages targeting low-income-earner emigrants’ households.

Furthermore, if emigration and remittances positively influence the social and economic determinants of health, including access to health care, it is worthwhile for governments in developing nations such as Zimbabwe to institute favourable international money transfer policies and systems. This includes revising the cost of remittance transfers downwards and effecting correct exchange rate regimes [9].

It is, however, necessary to note that there is a dearth of literature describing and explaining the emigration and health nexus. There is therefore need to replicate the study with larger populations and wider geographic coverage in order to allow for the comparison of communities with different socio-economic classes and to allow for broader generalisations.

### Limitations

This study was limited to one purposively selected district and a limited number of respondents from randomly selected households because of limited funds and time available. Secondly, the emigrants could not be interviewed; hence, findings were based on data from sending household respondents without the complementing data from the immigrants themselves.

## Data Availability

The data sets generated and/or analysed during the current study are not publicly available because individual privacy could be compromised, but are available from the corresponding author upon reasonable request.
